# Circulating plasma IL-13 and periostin are dysregulated type 2 inflammatory biomarkers in prurigo nodularis: A cluster analysis

**DOI:** 10.3389/fmed.2022.1011142

**Published:** 2022-12-06

**Authors:** Varsha Parthasarathy, Karen Cravero, Junwen Deng, Zhe Sun, Sarah M. Engle, Autum N. Auxier, Nathan Hahn, Jonathan T. Sims, Angela J. Okragly, Martin P. Alphonse, Shawn G. Kwatra

**Affiliations:** ^1^Department of Dermatology, The Johns Hopkins University School of Medicine, Baltimore, MD, United States; ^2^Lilly Research Laboratories, Eli Lilly and Company, Indianapolis, IN, United States

**Keywords:** pruritus, itch, prurigo, inflammation, IL-13, Th2 (type-2) immune responses

## Abstract

**Importance:**

Prurigo nodularis (PN) is a chronic heterogeneous inflammatory skin disease.

**Objective:**

To elucidate which components of type 2 inflammation are dysregulated systemically in PN.

**Design:**

Whole blood was obtained from PN patients with uncontrolled disease and control patients without pruritus. Plasma was assayed for IL-4, IL-5, IL-13, IgE, and periostin. ANOVA was utilized to compare PN and control patients and multiple-hypothesis adjusted *p-*value was calculated with the significance threshold at 0.05. Clustering was performed using K-means clustering.

**Participants:**

PN patients (*n* = 29) and controls (*n* = 18) from Johns Hopkins Dermatology had similar age sex, and race distributions.

**Results:**

Single-plex assays of the biomarkers demonstrated elevated circulating plasma IL-13 (0.13 vs. 0.006 pg/mL, *p* = 0.0008) and periostin (80.3 vs. 60.2 ng/mL, *p* = 0.012) in PN compared to controls. IL-4 (0.11 vs. 0.02 pg/mL, *p* = 0.30) and IL-5 (0.75 vs. 0.40 pg/mL, *p* = 0.10) were not significantly elevated, while IgE approached significance (1202.0 vs. 432.7 ng/mL, *p* = 0.08). Clustering of PN and control patients together revealed two clusters. Cluster 1 (*n* = 36) consisted of 18 PN patients and 18 controls. Cluster 2 (*n* = 11) consisted entirely of PN patients (*p* < 0.01). Cluster 2 had higher levels of IL-13 (0.33 vs. 0.008 pg/mL, *p* = 0.0001) and IL-5 (1.22 vs. 0.43 pg/mL, *p* = 0.03) compared to cluster 1.

**Conclusion and relevance:**

This study demonstrates elevation of IL-13 and periostin in the blood of PN patients, with distinct clusters with varying degrees of type 2 inflammation. Given this heterogeneity, future precision medicine approaches should be explored in the management of PN.

## Introduction

Prurigo nodularis (PN) is a chronic inflammatory skin disease that presents with extremely itchy, hyperkeratotic nodules on the extremities and trunk, dramatically reducing quality of life for patients to a level similar to conditions such as stroke and heart failure (1). Recent studies have identified increased CD4^+^, CD8^+^, γδ, and natural killer T cells in circulating peripheral blood mononuclear cells (PBMCs) of PN patients, suggesting PN is associated with systemic inflammation (2). There is also evidence of varying levels of T-helper (T_*h*_)1, T_*h*_2, T_*h*_17, and T_*h*_22 involvement in both the skin and blood of PN patients, suggesting a role for multiple immune pathways in its pathogenesis (2, 3). The systemic inflammatory impact of PN is supported by its extensive comorbidity burden, such as chronic kidney disease, type 2 diabetes mellitus, and malignancy (4, 5).

The role of type 2 inflammation is of interest in PN, given that there have been several off-label reports of the efficacy of treatment response with dupilumab, an IL-4 receptor α inhibitor that prevents IL-4 and IL-13 signaling (6). There are a number of pruritic skin conditions associated with Type 2 inflammation, including atopic dermatitis, bullous pemphigoid, urticaria, and parasitic diseases (7). However, PN is unique from classical type 2 inflammatory diseases, such as atopic dermatitis, because it features a novel clinical presentation and unique neurovascular and fibrotic gene dysregulation, with upregulation of axon regeneration and vascular endothelial growth factor activity (8). There is also preliminary evidence of disease heterogeneity in clinical presentation and systemic inflammation, with suggestions of neural- and immune-biased disease endotypes (9). For example, in a study of plasma biomarkers, an inflammatory subset of prurigo patients presented with higher levels of interleukin (IL)-1α, IL-4, IL-5, IL-6, IL-10, IL-17A, IL-22, IL-25, and interferon (IFN)-α (9). Additionally, there are numerous comorbidity profiles that support disease heterogeneity in PN, including a subset of patients who present with atopic comorbidities, while others present with neurovascular comorbidities (10). Therefore, here we elucidate which components of type 2 inflammation are dysregulated in PN patients and test the hypothesis that there are distinct patient subgroups with varying degrees of involvement of type 2 immunity among PN patients.

## Methods

Whole blood was obtained from PN patients with uncontrolled disease for at least 6 weeks (defined as a Worst Itch Numeric Rating Scale score of ≥ 7 out of 10 points and nodule count of ≥ 20 nodules) and control patients without pruritus (0 out of 10 score on Worst Itch Rating Scale) under a Johns Hopkins IRB-approved study at the Johns Hopkins Outpatient Center dermatology clinic (IRB00231694) (11). Effect size was determined using other papers examining cytokines in pruritic skin diseases. Power calculations were performed with the following parameters: power of 90%, two-tailed alpha of 0.05, and estimated effect size of 1.5–2 for the biomarkers yielded 10 patients per group required to perform analyses (9).

All PN patients met formal criteria for prurigo as determined by an expert consensus panel, which includes: the presence of nodular lesions; chronic pruritus for at least 6 weeks; and a history or signs of repeated scratching, picking, or rubbing (10, 11). All PN patients also had an Investigator Global Assessment score of severity of ≥ 3. Control patients presented to clinic for: full body skin exams (5/18, 28%), non-inflammatory benign skin conditions such as warts and skin tags (5/18, 28%), localized skin conditions such as acne vulgaris and seborrheic dermatitis (4/18, 22%), or as previously identified healthy controls (4/18, 22%). Atopic predisposition for all patients was identified as presence of 2 out of 3 of the following atopic conditions as per previous studies: asthma, allergic rhinitis, or atopic dermatitis (12).

Plasma samples were isolated from the whole blood by centrifugation for 10 min at 1960 xg. Plasma was then carefully separated, avoiding the buffy coat, and transferred into separate tubes to create 1 mL aliquots, with at least 4 aliquots isolated. Next, the aliquots were slowly frozen at a temperature of -80°C for 24–72 h. Plasma was then assayed for IL-4, IL-5, IL-13, immunoglobulin E (IgE), and periostin. IL-4 and IL-5 were measured with S-PLEX Human IL-4 and IL-5 kits (MSD, Rockville, MD). IL-13 was measured using the Simoa^®^ human IL-13 Advantage HD-1/HD-X kit (Quanterix, Billerica, MA). IgE was measured with Invitrogen human IgE ELISA kit and periostin was assayed with the Invitrogen human periostin ELISA kit (ThermoFisher Scientific, Frederick, MD). For statistical analysis, analysis of variance (ANOVA) was utilized to compare PN and control patients using log-transformed data. For between-markers multiplicity adjustment, adjusted *p-*value was calculated with a Benjamini-Hochberg procedure with the significance threshold at 0.05. Clustering of PN and control patients was performed using K-means clustering algorithm in R version 4.0.3, and differences in markers between clusters was calculated using ANOVA.

## Results

**FIGURE 1:**
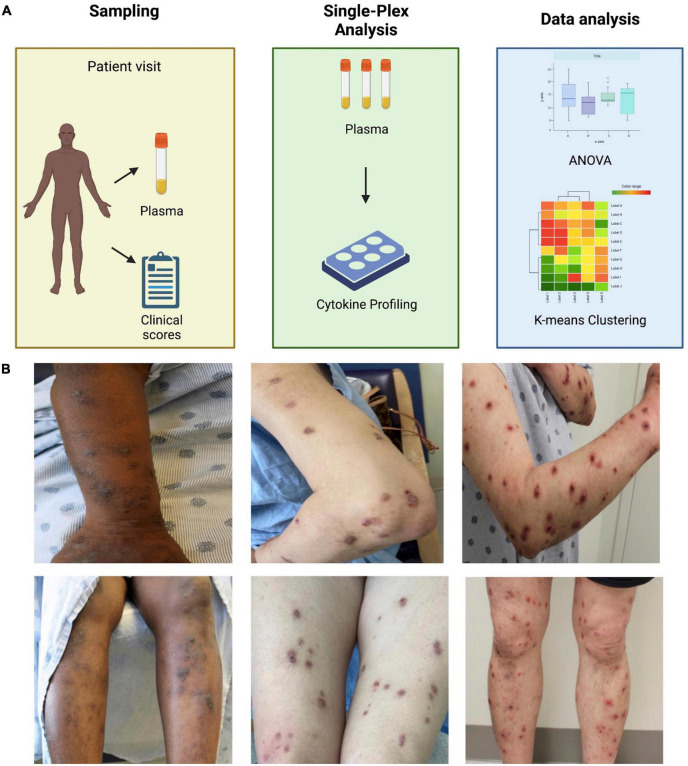
**(A)** Schematic of methods. **(B)** Representative images of prurigo nodularis patients.

PN patients (*n* = 29) and controls (*n* = 18) had similar age (53.8 ± 13.4 vs. 52.2 ± 13.2 years, *p* = 0.68), sex (72% vs. 72% female, *p* = 0.98), and race (21% vs. 33% Caucasian and 69% vs. 67% African American, *p* = 0.46) distributions. PN patients had higher Worst Itch Numeric Rating Scale scores (8.9 ± 1.3 vs. 0 ± 0) and PN Investigator Global Assessment scale scores (3.3 ± 0.6 vs. 0 ± 0) compared to controls. Seven PN patients had evidence of concomitant atopic dermatitis or history of atopic dermatitis, while zero controls had atopic history. Baseline demographics are outlined in Table 1. Methodology and representative images of PN patients are shown in Figure 1.

**TABLE 1:** Baseline demographics of patients.

Characteristics	PN (*n* = 29)	Controls (*n* = 18)	*P-*value
Age (mean ± SD)	53.8 ± 13.4	52.2 ± 13.2	0.68
Gender			0.98
Female (*n*, %)	21 (72)	13 (72)	
Male (*n*, %)	8 (28)	5 (28)	
Race			0.55
White or Caucasian (*n*, %)	6 (21)	6 (33)	
Black or African American (*n*, %)	20 (69)	12 (67)	
Asian (*n*, %)	2 (7)	0 (0)	
Other (*n*, %)	1 (3)	0 (0)	
Ethnicity			1.00
Hispanic and/or Latino (*n*, %)	0 (0)	0 (0)	
Not Hispanic and/or Latino (*n*, %)	29 (100)	18 (100)	
Worst Itch Numeric Rating Scale score (mean ± SD)	8.9 ± 1.3	0 ± 0	<0.001
Investigator’s Global Assessment (mean ± SD)	3.3 ± 0.6	0 ± 0	<0.001

PN, prurigo nodularis; SD, standard deviation.

**FIGURE 2:**
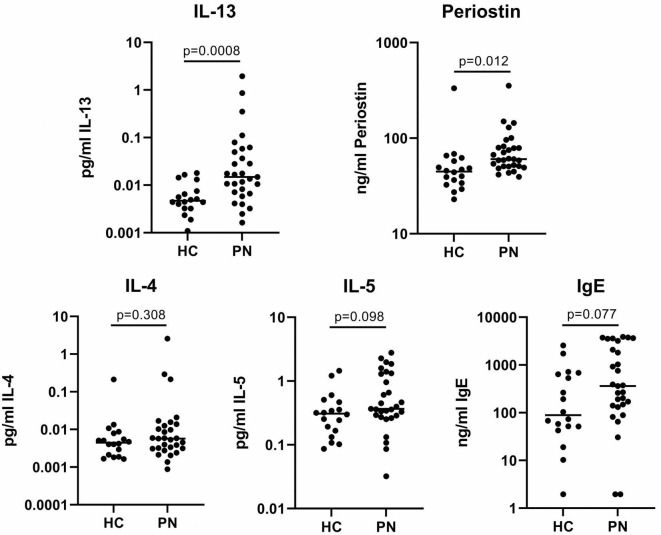
Plasma single-plex immunoassays. PN, prurigo nodularis; HC, healthy control.

Single-plex assays of the type 2 inflammatory biomarkers demonstrated significantly elevated circulating plasma IL-13 (0.13 vs. 0.006 pg/mL, *p* = 0.0008) and periostin (80.3 vs. 60.2 ng/mL, *p* = 0.012) in PN patients compared to controls. IL-4 (0.11 vs. 0.02 pg/mL, *p* = 0.30) and IL-5 (0.75 vs. 0.40 pg/mL, *p* = 0.10) were not significantly elevated, while IgE approached significance in PN (1202.0 vs. 432.7 ng/mL, *p* = 0.08) (Figure 2).

**FIGURE 3:**
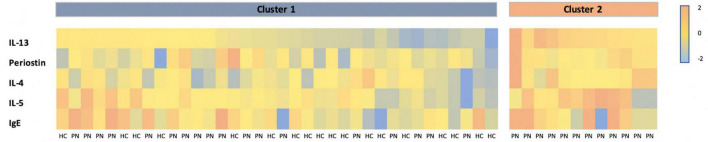
Heatmap of *Z*-scored biomarker levels for each patient, delineated by cluster. PN, prurigo nodularis; HC, healthy control.

K-means clustering of PN and control patients together revealed mean silhouette maximization at two clusters. Cluster 1 (*n* = 36) consisted of 18 PN patients and 18 controls. Cluster 2 (*n* = 11) consisted entirely of PN patients (*p* < 0.01) (Figure 3). When comparing the two clusters, cluster 2 had higher levels of IL-13 (0.33 vs. 0.008 pg/mL, *p* = 0.0001) and IL-5 (1.22 vs. 0.43 pg/mL, *p* = 0.03) compared to cluster 1. There were no differences in periostin (*p* = 0.06), IL-4 (*p* = 0.07), or IgE (*p* = 0.41) between these groups. PN patients from cluster 2 (*n* = 11) demonstrated higher levels of IL-13 than cluster 1 PN patients (0.33 pg/mL in cluster 2 vs. 0.007 pg/mL in cluster 1, *p* = 0.0003). There were no significant differences in the biomarkers between PN patients and healthy control patients in cluster 1.

## Discussion

Here we find two components of type 2 inflammation, IL-13 and periostin, are increased in the circulating blood of PN patients. IL-13 is also increased in atopic dermatitis and is a potent enhancer of neuronal responses of pruritus and neurogenic inflammation (13, 14). Periostin is an extracellular matrix protein located in the dermis that is involved in type 2 inflammatory responses and is correlated with progressive sclerosis in systemic sclerosis (15). Additionally, the PN and healthy populations examined have similar distributions of age, sex, and race in PN vs. healthy populations, as age may play a role in serum periostin levels, with younger patients having higher serum periostin (16). The upregulation of these markers also highlights the role of the neuroimmune axis in PN, as both IL-13 and periostin directly stimulate nerve fibers via receptor alpha_*v*_beta_3_ (17). Furthermore, IL-13 and periostin both stimulate the release of the highly pruritic cytokine IL-31 from inflammatory cells, including macrophages and eosinophils, propagating systemic inflammation in PN (18, 19). IL-31 is a known marker of itch in type 2 inflammatory diseases such as atopic dermatitis and PN (20, 21). The role of IL-31 in PN and atopic dermatitis has been investigated. IL-31 directly interacts with sensory neurons, activates granulocytes, and influences transcriptomic profiles of epidermal keratinocytes. Therefore, IL-31 is a compelling target for treatment, with current trials for nemolizumab (a monoclonal antibody targeting IL-31 receptor alpha) (22, 23). Since the importance of IL-31 has been described, this paper aimed to highlight other Th2-associated markers, such as periostin, that may be involved in the pathogenesis of PN.

Of note, there were no differences in periostin levels between the two clusters. As periostin may play numerous roles in the pathogenesis of itch, both neuropathic and inflammatory, there may be different mechanisms by which periostin exacerbates pruritus in PN. Additionally, IL-5 was identified as a marker that was higher in the more inflammatory cluster. IL-5 is a potent regulator of genes involved in growth, survival, and effector function of eosinophils (24). PN, which in subsets of patients is associated with increased blood eosinophils, has also been associated with higher levels of major basic protein and eosinophil-derived neurotoxin in some patients (25).

Previous studies have suggested endotypes in PN, represented by varying degrees of circulating inflammatory biomarkers across varying immune axes, including plasma IL-1α, IL-4, IL-5, IL-6, IL-10, IL-17A, IL-22, IL-25, and IFN-α (9). Our findings provide further evidence of distinct endotypes in PN with respect to type 2 inflammation, with one cluster consisting solely of PN patients with elevated IL-13 compared to another less inflammatory cluster without elevated IL-13. These results showed no significant differences between the non-inflammatory subtype of PN and control patients with respect to type 2 inflammatory biomarkers. This supports clinical observations of heterogeneity in response to type 2 inflammatory-modulating therapies in PN patients (6). Additional evidence of varying degrees of type 2 inflammatory dysregulation clinically includes highly variable levels of blood eosinophils and IgE, which serve as predictors of response to immunomodulatory therapy in other chronic pruritic conditions such as chronic pruritus of unknown origin (26, 27).

Limitations of this study include a cross-sectional design and small sample size which may prevent inference of causation. Results of this study demonstrate elevation of IL-13 and periostin in PN patients with distinct clusters of varying degrees of type 2 inflammation, supporting prior findings of disease heterogeneity in PN. The identification of endotypes with varying degrees of inflammatory axis signaling may help explain varying differences in phenotypic presentation observed between patients. Given the diversity of this disease, further biomarker studies may help elucidate future precision medicine approaches in the management of PN.

## Data availability statement

The raw data supporting the conclusions of this article will be made available by the authors, without undue reservation.

## Ethics statement

The studies involving human participants were reviewed and approved by the Johns Hopkins Institutional Review Board. The patients/participants provided their written informed consent to participate in this study. Written informed consent was obtained from the individual (s) for the publication of any potentially identifiable images or data included in this article.

## Author contributions

MA, SK, JS, and AO contributed to the conception and design of the study. VP collected the samples, assisted in data analysis, and wrote the manuscript draft. KC and JD assisted in data analysis and manuscript drafting. ZS, AA, NH, and SE helped acquire and analyzed the data. All authors contributed to the manuscript revision, read, and approved the submitted version.
